# Healthcare-associated links in transmission of nontuberculous mycobacteria among people with cystic fibrosis (HALT NTM) study: Rationale and study design

**DOI:** 10.1371/journal.pone.0261628

**Published:** 2021-12-20

**Authors:** Jane E. Gross, Silvia Caceres, Katie Poch, Nabeeh A. Hasan, Rebecca M. Davidson, L. Elaine Epperson, Ettie Lipner, Charmie Vang, Jennifer R. Honda, Matthew Strand, Michael Strong, Lisa Saiman, D. Rebecca Prevots, Kenneth N. Olivier, Jerry A. Nick

**Affiliations:** 1 Department of Pediatrics, National Jewish Health, Denver, CO, United States of America; 2 Department of Medicine, National Jewish Health, Denver, CO, United States of America; 3 Center for Genes, Environment and Health, National Jewish Health, Denver, CO, United States of America; 4 Division of Biostatistics, National Jewish Health, Denver, CO, United States of America; 5 Department of Pediatrics, Columbia University Irving Medical Center, New York, NY, United States of America; 6 Laboratory of Clinical Infectious Diseases, National Institute of Allergy and Infectious Diseases, NIH, Bethesda, MD, United States of America; 7 Pulmonary Branch, National Heart, Lung, and Blood Institute, NIH, Bethesda, MD, United States of America; Public Health England, UNITED KINGDOM

## Abstract

**Background:**

Healthcare-associated transmission of nontuberculous mycobacteria (NTM) among people with cystic fibrosis (pwCF) has been reported and is of increasing concern. No standardized epidemiologic investigation tool has been published for healthcare-associated NTM outbreak investigations. This report describes the design of an ongoing observational study to standardize the approach to NTM outbreak investigation among pwCF.

**Methods:**

This is a parallel multi-site study of pwCF within a single Center who have respiratory NTM isolates identified as being highly-similar. Participants have a history of positive airway cultures for NTM, receive care within a single Center, and have been identified as part of a possible outbreak based on genomic analysis of NTM isolates. Participants are enrolled in the study over a 3-year period. Primary endpoints are identification of a shared healthcare-associated encounter(s) among patients in a Center and identification of environmental isolates that are genetically highly-similar to respiratory isolates recovered from pwCF. Secondary endpoints include characterization of potential transmission modes and settings, as well as incidence and prevalence of healthcare-associated environmental NTM species/subspecies by geographical region.

**Discussion:**

We hypothesize that genetically highly-similar strains of NTM among pwCF cared for at the same Center may arise from healthcare sources including patient-to-patient transmission and/or acquisition from environmental sources. This novel study design will establish a standardized, evidence-based epidemiologic investigation tool for healthcare-associated NTM outbreak investigation within CF Care Centers, will broaden the scope of independent outbreak investigations and demonstrate the frequency and nature of healthcare-associated NTM transmission in CF Care Centers nationwide. Furthermore, it will provide valuable insights into modeling risk factors associated with healthcare-associated NTM transmission and better inform future infection prevention and control guidelines. This study will systematically characterize clinically-relevant NTM isolates of CF healthcare environmental dust and water biofilms and set the stage to describe the most common environmental sources within the healthcare setting harboring clinically-relevant NTM isolates.

**Trial registration:**

ClinicalTrials.gov NCT04024423. Date of registry July 18, 2019.

## Introduction

Pulmonary nontuberculous mycobacteria (NTM) is one of the most challenging infections to treat among people with cystic fibrosis (pwCF), notable for prolonged antibiotic courses and often poor response to therapy [1, 2]. Positive cultures for NTM occur in about 20% of pwCF over a 5-year period [3]. However, source(s) of infection, modes of transmission, and exposure risks are poorly understood [4]. It is thought that NTM are primarily acquired from environmental sources including soil, water, water supply systems, and from aerosols generated by flowing water [5–7]. Nonetheless, no direct molecular link has been established between environmental NTM and respiratory CF NTM [8]. Healthcare-associated transmission of NTM among pwCF is of growing concern worldwide [9–12]. One study reported widespread global transmission of NTM, potentially via person-to-person transmission of fomites and aerosols [13]. Despite reports of NTM outbreaks in CF Care Centers, no standardized epidemiologic investigation tool for healthcare-associated NTM acquisition has been published. Criteria are needed to validate NTM transmission with genetically-matched isolates between patients and/or environment and evidence of epidemiologic exposure with potential cross-infection [14]. Whole genome sequencing (WGS) is the gold standard for isolate comparison and is utilized in numerous respiratory CF NTM outbreak reports supporting *Mycobacterium abscessus* transmission within CF Centers [10, 12, 13, 15], as well as in reports refuting transmission of *M*. *abscessus* among pwCF [16, 17].

NTM can colonize municipal water systems and have been identified in healthcare facilities [18–20]. NTM are found in water and in biofilms on water supply system pipes, with both being implicated in healthcare-associated outbreaks of infection [21]. A multicenter prevalence study of NTM found no nosocomial transmission among pwCF, and molecular analysis at that time revealed almost all patients had unique NTM strains [22]. Another study described a biphasic nosocomial outbreak of *M*. *abscessus* linked to hospital tap water [23]. The first phase outbreak of respiratory *M*. *abscessus* was attributed to routine care practices using hospital tap water with exposure to the aerodigestive tract in high-risk patients. The second phase outbreak among cardiac surgery patients with invasive *M*. *abscessus* infections was attributed to contaminated heater-cooler units of cardiopulmonary bypass machines. In other settings, inhalation of aerosolized NTM via showerheads is a possible source of acquisition [24], where municipal water harbor clinically-relevant NTM [25].

The Colorado National Resource Centers (CO-NRC) serves as a national reference biorepository for CF NTM [26]. Respiratory isolates received from Care Centers across the U.S. were submitted and collected to study CF-associated NTM genetics. Samples were banked and underwent WGS [26]. WGS analysis identified clusters of NTM isolates, defined as highly similar strains at the genomic level, harbored by two or more pwCF who are cared for at the same Center. These identifications heightened our concern for potential healthcare-associated NTM acquisition originating from patient-to-patient transmission or a common environmental source(s) within U.S. CF Care Centers. This is the first study to standardize healthcare-associated outbreak investigation of NTM among pwCF cared for at a single CF Care Center (Table 1).

**Table 1:** Study objectives.

**Primary Objectives**	1. Epidemiologic investigation: Facilitate implementation of a standardized process by which individual CF Care Centers perform an epidemiologic investigation of NTM clusters as identified by the CO-NRC.
	2. Environmental investigation: Determine if NTM strains identified in clusters are genetically related to strains isolated from dust and/or water biofilm sources within the CF healthcare setting.
**Secondary Objectives**	1. Determine points of potential transmission settings: Frequency, location and source of patient overlap within the healthcare system within the 2-year period prior to the detection of NTM colonization.
	2. Evaluate infection prevention and control (IP&C) measures: Use of single patient rooms, healthcare staff gowning and gloving, patients wearing masks, cleaning procedures between patients, and maintenance of patient-to-patient separation of 6 feet or more within the 2-year study period.
	3. Culture NTM isolates from dust and water biofilms collected from the healthcare environment.

## Materials and methods

### General overview

**Fig 1:**
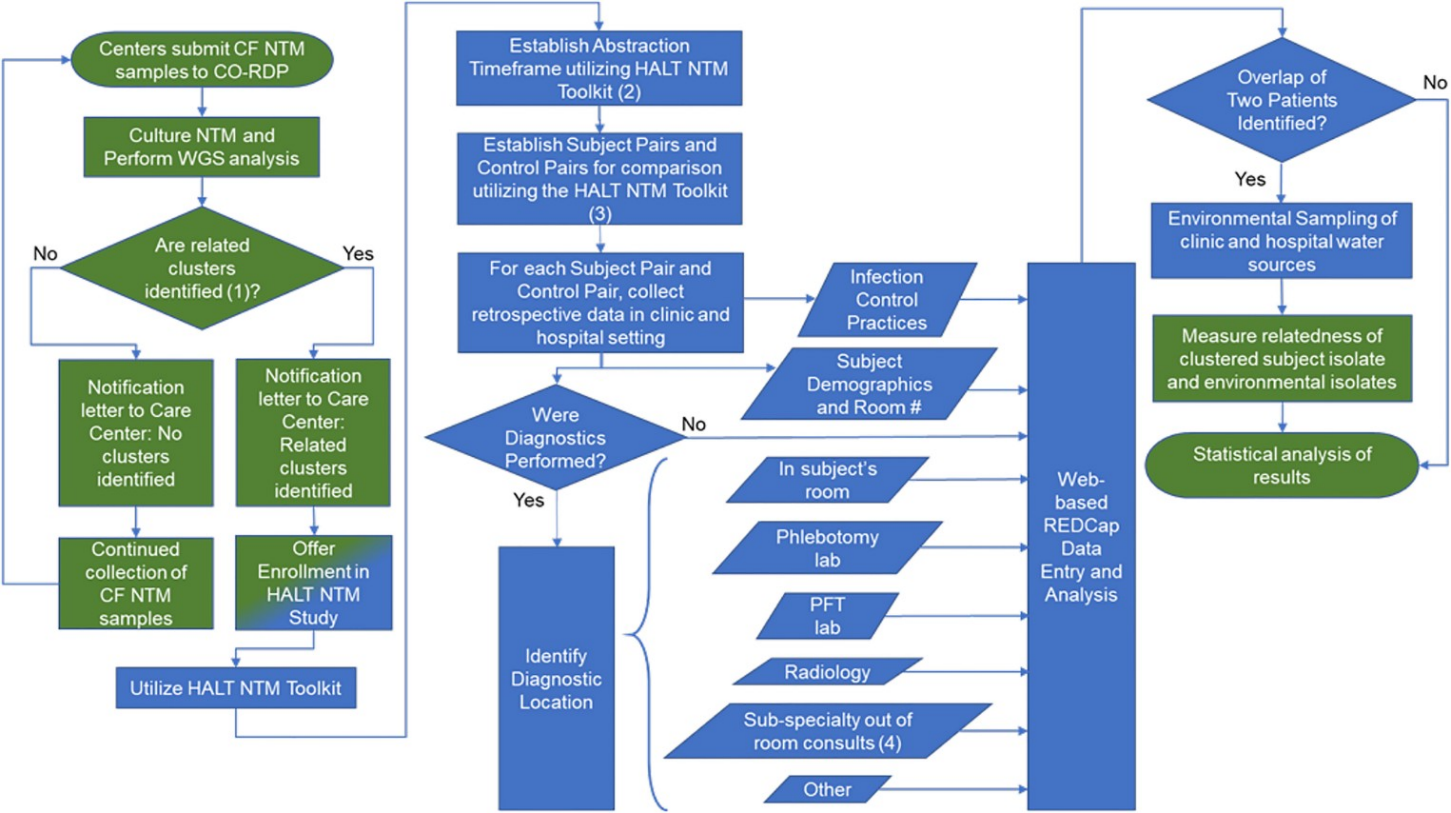
Schematic showing an overview of the healthcare-associated links in transmission of nontuberculous mycobacteria among people with cystic fibrosis (HALT NTM) study.

**Fig 2:**
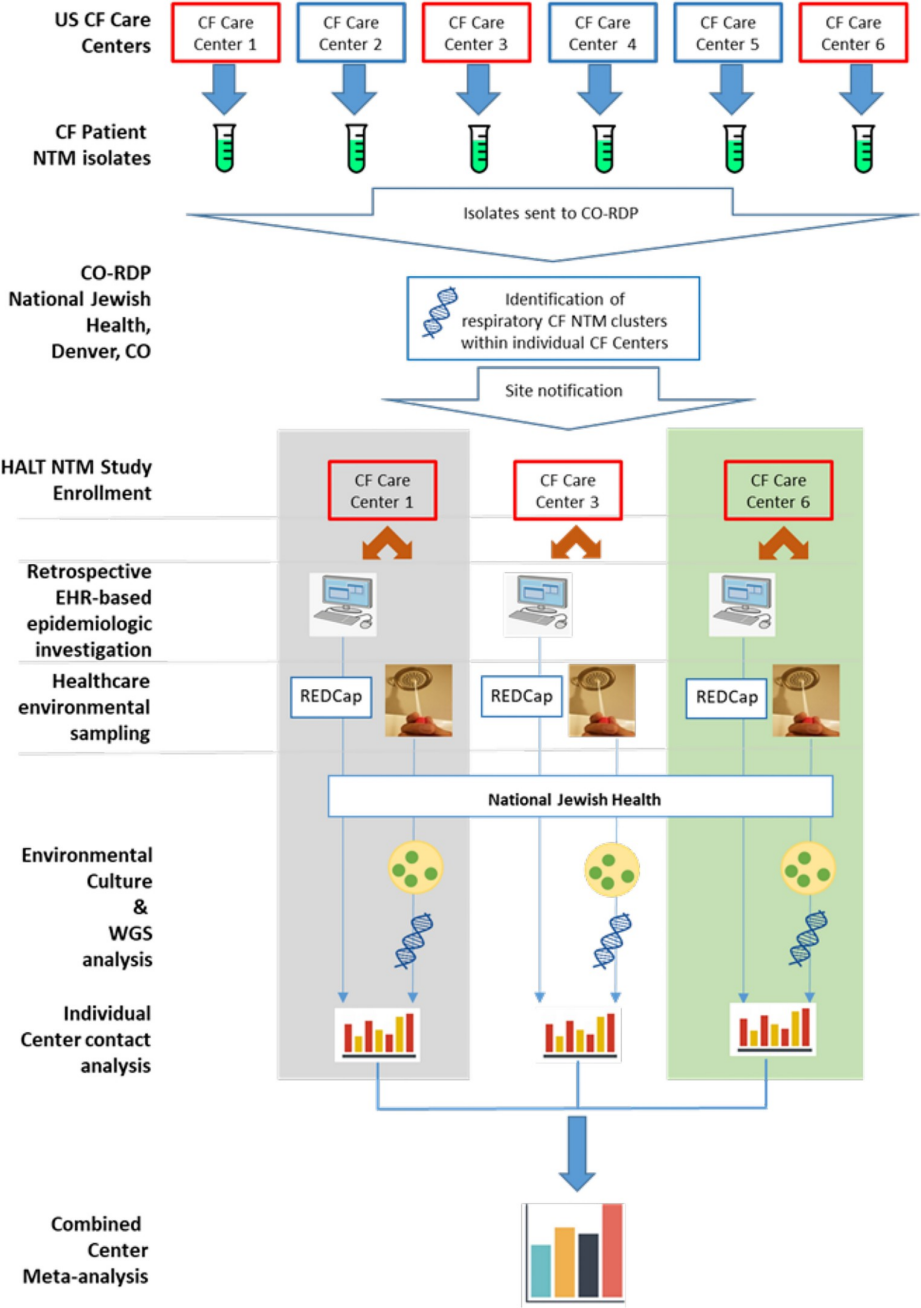
Schematic showing an overview of the parallel multi-site study design.

This is a parallel multi-site study of pwCF within a single CF Center who have respiratory NTM isolates identified in clusters. An overview of the Healthcare-Associated Links in Transmission of NTM among pwCF (HALT NTM) investigation process is outlined in Fig 1 (schematic showing an overview of the study design) and the parallel multi-site study concept is outlined in Fig 2 (parallel multi-site study overview). Clusters are defined as highly similar strains at the genomic level, harbored by two or more pwCF who are cared for at the same CF Care Center. We are using the CO-NRC WGS data to identify clusters and inform CF Care Centers of NTM clusters at their Center [27, 28]. National Jewish Health Intuitional Review Board initially approved this study (#HS-3175-528) on October 9, 2018 and renewed approval on October 4, 2019. Biomedical Research Alliance of New York (BRANY) Intuitional Review Board approved this protocol (#HS-3175-528) on July 1, 2020 and renewed approval on Aug 17, 2021. Patient consent is not required for this study.

The purpose of the HALT NTM toolkit is to provide resources to systematically collect data on clustered CF NTM patients in order to determine if the source of NTM infection may be a healthcare-associated outbreak. We integrated clinical and epidemiological research methods to adapt a Centers for Disease Control and Prevention (CDC) standardized, and validated Healthcare-Associated Infection Outbreak Investigation Toolkit to systematically collect data for healthcare-associated NTM outbreak investigations [29]. Through consultation with subject matter experts in the field and an appropriate scientific literature review, we modified the CDC Healthcare-Associated Infection Outbreak Investigation Abstraction Form, which was designed to be utilized in local investigations of common healthcare-associated infections [30] to develop the HALT NTM Outbreak Investigation Abstraction Form. The HALT NTM toolkit is a web-based, REDCap®, branching logic questionnaire that is to be completed by a local CF Center team member. Using integrated clinical and epidemiological research methods, the HALT NTM retrospective epidemiologic investigation seeks to identify overlaps in space and time through mapping of visits and source(s) of care among patients with highly similar NTM isolates in a Center. The HALT NTM toolkit facilitates a standardized, stepwise process by which individual Centers perform retrospective epidemiologic evaluation of patients identified by the CO-NRC as part of an NTM cluster. A control comparison group is also included in the overlap analysis and is defined as pwCF cared for at the same Care Center identified with respiratory NTM that is not part of a cluster. The CO-NRC provides expertise to support Centers while implementing the investigation. Through a collaborative agreement, HALT NTM is available to the entire CF Foundation Care Network to conduct a standardized, independent, confidential NTM outbreak investigation.

In addition to the epidemiologic investigation, the healthcare environment is also sampled. Since clustered NTM isolates could originate from a shared healthcare source, biofilms from healthcare water supplies will be collected, in addition to dust samples from the same environment. NTM will be recovered, identified, and sequenced as previously described [7] to determine if the respiratory CF NTM strain genotype is similar to those recovered from the healthcare environment.

### Study population

The CO-NRC National Reference Biorepository for CF NTM receives respiratory isolates from CF Care Centers around the U.S. for the purpose of culture, molecular identification, and WGS. Using phylogenomic analysis of the core genome, the CO-NRC identifies clusters of NTM isolates, defined as highly similar strains at the genomic level, harbored by two or more pwCF who are cared for at the same CF Care Center [27, 31]. Highly-related clusters of NTM are defined as less than or equal to 20 or 30 single-nucleotide polymorphism (SNP) differences at the core genome level, for *M*. *avium* and *M*. *abscessus*, respectively. All CF Care Centers that have clusters of highly-related NTM isolates are offered enrollment in HALT NTM. Subject enrollment is unlimited and based on the number of pwCF identified in an NTM cluster(s) within an individual CF Care Center (prospectively collected data). The inclusion and exclusion criteria are shown in Table 2.

**Table 2:** Inclusion and exclusion criteria.

Inclusion Criteria	Exclusion Criteria
1. Written informed consent is not required for this retrospective epidemiological study.	1. No formal diagnosis of CF.
2. Diagnosis of CF consistent with the 2017 CFF guidelines [32].	2. A patient not followed at a U.S. CF Care Center.
3. Male or female pwCF of any age who has a history of NTM species or sub-species collected from expectorated sputum, induced sputum and/or bronchoalveolar lavage and identified by the CO-NRC as falling into a highly-related cluster within a single CF Care Center.	

### Measures

The following baseline demographic and clinical characteristics will be reported for each subject: age, sex, race, ethnicity, zip code, and NTM species/subspecies.

The HALT NTM abstraction toolkit will be used to assess if an overlap occurred between two or more pwCF identified in a cluster within the CF healthcare setting, defined as CF clinic, hospital, and research settings. An overlap is defined as a healthcare setting where two or more pwCF identified in the cluster received care in the same setting for any period of time within 24 hours. A point instance of overlap is defined as a specific physical space within the healthcare setting (clinic, hospital, or research setting) where two or more pwCF identified in the cluster received care.

To evaluate all potential patient overlaps, patients within the identified cluster will be compared in pairs based on date of first positive NTM culture. The range of dates required for data collection between two patients is called the exposure window and is determined using the abstraction tool within the HALT NTM toolkit. Abstraction is based on Subject 1 being identified as the subject with the longest colonization of NTM and Subject 2 being identified as the subject with the second longest colonization of NTM. The start date for the exposure window is 2 years prior to the date that the highly-related NTM isolate was first recovered in Subject 2. The clinical significance of a first positive CF NTM culture among subjects that develop active NTM disease is demonstrated in lower baseline forced expiratory volume in 1 second (FEV_1_) at the time of first positive culture and an increased rate of decline in FEV_1_ one year preceding the first positive culture [33]. Based on this finding, it is reasonable to infer that NTM acquisition may occur up to 2 years prior to first positive culture. The end date for the exposure window is the date of sample collection for the highly-related strain that was isolated from Subject 2. With this information, a directionality of possible transmission from Subject 1 to Subject 2 is established, regardless of how many patients are identified in the cluster. Subject and control pairs are established using the patient-pairing tool within the HALT NTM toolkit. The number of paired comparisons depends on the total number of subjects in the cluster, i.e., 2 subjects = 1 pair for comparison; 3 subjects = 3 pairs for comparison; 4 subjects = 6 pairs for comparison, etc.

The control group will consist of pwCF at the same institution that are NTM positive for the same species/subspecies as the identified cluster, but have distinctly different isolates at the genomic level. Once subject pairs are established within the toolkit, a retrospective electronic health record (EHR) chart review of clinic, hospitalizations, and research visits will be used to collect data. Requested data will be documented within the web-based HALT NTM toolkit. Information collected will include subject demographics, room numbers, policy information on infection prevention and control (IP&C) practices, and details on procedures and subject location throughout the healthcare interaction. Specific details will include location where phlebotomy, spirometry, imaging, consults, and other healthcare-associated interactions occur. Pairwise assessment of opportunities for transmission events in the healthcare setting and longitudinal NTM culture results will be determined within the abstraction timeframe.

The HALT NTM toolkit will be completed by a local CF Center team member familiar with local CF care processes, terminology, and chart review. We strongly encourage involvement of the local IP&C team. The abstracted data will be directly entered into the HALT NTM toolkit and stored in a central REDCap® database on a server maintained by National Jewish Health (NJH). Data, including protected health information (PHI), entered by a Center will only be visible to that Center and study consultants at NJH. PHI is de-identified for analysis. Fig 1 highlights the data collection process.

This study will also collect and analyze dust and water biofilms from a variety of environmental sources in the healthcare setting including vents, sink faucets, showerheads, shower hoses, ice machines, drinking fountains, patient-utilized coffee machines, and decorative water features in the clinic, hospital, and research settings in which pwCF receive care. The number of environmental samples collected per room will depend on the number of water biofilm surfaces in the room. Generally, one water biofilm sample will be obtained from each water source in a room and one dust sample will be obtained from each room. In a typical clinic or research space, one sample will be obtained from the sink faucet and one dust sample from a vent. In a typical hospital room, one sample will be obtained from each sink faucet (patient room and patient bathroom), one sample will be obtained from the shower head and hose or bath faucet, and one dust sample from a vent. In common spaces within the healthcare system, one sample will be obtained from each water source including water dispensers, ice machines, drinking fountains, patient-utilized coffee machines, and decorative water features as well as one dust sample from a vent. Environmental healthcare-associated dust and water biofilms will be selectively cultured for NTM using standard microbiological approaches as described [34]. Genomic DNA will be extracted from bacterial pellets [35] and isolates recovered will be identified to the species or subspecies level through amplification and sequencing of the RNA polymenrase beta subunit (*rpo*B) gene as described [7, 34, 36]. Clinically-relevant NTM that are also isolated from pwCF will be further analyzed by WGS.

### Outcomes

#### Primary outcome

The primary endpoint of the epidemiologic investigation identifies the potential transmission settings and quantifies the probability of healthcare-associated patient-to-patient transmission of NTM within individual CF Care Centers. We further characterize the source(s) of direct or indirect transmission of NTM within the individual CF healthcare setting.

The primary endpoint of environmental sampling of dust and water biofilm collection characterizes clinically-relevant NTM found within CF healthcare settings and determines if the environmental isolates are genetically similar to isolates recovered from patients.

#### Secondary outcome

Secondary endpoints include: 1) Characterization of CF Care Center adherence to the CF IP&C guidelines [37] and potential transmission settings and exposures. Basic summaries of points of potential transmission settings are provided. 2) Incidence and prevalence of NTM species and subspecies within healthcare dust and water biofilms are characterized. 3) Identification of NTM isolates in dust and biofilms, and comparison with highly-related patient isolates.

### Quality assurance and monitoring

#### Quality assurance

After data have been entered into the study database, data validation checks will be applied on a regular basis. As part of data analysis, the lead biostatistician on the study will monitor for data outliers and perform data validation. If data entry errors are encountered, the study database will be updated in accordance with the resolved queries. All changes to the study database will be documented in an audit trail.

#### Monitoring

The study Principal Investigator (PI) and the CF Foundation are authorized to monitor the study. By signing the protocol, the Investigator grants permission to the Sponsor (or designee), and appropriate regulatory authorities to conduct on-site monitoring and/or auditing of all appropriate study documentation. Monitoring visits are conducted by representatives of the Sponsor according to the U.S. CFR 21 Part 312 and ICH Guidelines for Good Clinical Practice (GCP) (E6) to ensure investigator compliance to 21 CFR Parts 50, 56 and 312 and to GCP.

The Investigator must make study data accessible to the monitor, other authorized representatives of the Sponsor (or designee), Institutional Review Board/Independent Ethics Committee, and Regulatory Agency (e.g., FDA) inspectors upon request. Hard copy files are not created. All study information is maintained in REDCap® for a period of 5 years after database lock. There may be other circumstances for which the Sponsor is required to maintain study records and, therefore, the Sponsor should be contacted prior to removing study records for any reason.

### Data management and data analysis

#### Data management

Study personnel at each site will enter data from the EHR and CFF Patient Registry corresponding to a participant’s visit into the protocol-specific electronic REDCap® database when the information corresponding to that visit is available. Participants will not be identified by name in the study database to be collected by the Sponsor (or designee), but will be identified by a site number, participant number and initials. For all REDCap® data entry, the time and date stamp will track the person entering or updating data and create an electronic audit trail. The Investigator will be responsible for all information collected on participants enrolled in this study. All data collected during the course of this study must be reviewed and verified for completeness and accuracy by the Investigator. At the completion of the study, a copy of the site-specific REDCap® data will be provided to the site to be retained at the Investigator’s site.

REDCap® is a secure, HIPAA compliant, web-based application designed to support data capture for research studies. REDCap® is maintained by the REDCap® Consortium which comprises over 3,500 institutional partners including NJH, and is administrated locally by the NJH Research Informatics Services. Access to the database requires user authentication with password. All data are stored on a secure server, and backups are encrypted. The NJH Research Informatics Services Core is used as a central location for data processing and management.

#### Sample size

All U.S. CF Care Centers are eligible for enrollment. The sample size of each cluster, and the number of clusters analyzed, depends on the number of pwCF attending the Center, the number with a history of NTM infection, and the number of available respiratory NTM isolates provided for WGS analysis and clusters identified within each Center. If in fact an outbreak event occurred within a Center, it is likely that the size of each cluster would be larger. As such, sample size will vary with each Center.

#### General analysis plan

All analyses are based on the group of subjects that are identified by the CO-NRC as having genetically similar NTM isolates. The epidemiologic and environmental sampling data from each CF Care Center will be analyzed independently. A timeline for completion of data collection and analysis for a participating Center is shown in Table 3. Upon completion of multi-site enrollment, the aggregate results of epidemiologic investigation and environmental sampling from individual CF Care Centers will undergo meta-analysis as seen in Fig 2.

**Table 3:** Timeline of data collection and analysis.

Week(s)	1–4	5	7	9–12	13–15	16–18	19–22	23–24
	Local site epidemiologic investigation	Receipt of Environmental samples at NJH	Initial plating of environmental samples	Culture NTM on solid media	Inoculate colony in broth	Sequence DNA and identify NTM isolate	Perform WGS and data analysis	Report results to CF Center

#### Primary analysis

For the primary endpoint, analysis of the individual center individual data will be completed using both qualitative and quantitative approaches. We will identify a set of variables used to assess degree of proximity of subjects in time and/or space. The first analysis will involve quantifying exposure windows for patients within clusters at a site based on hospital or clinic visits, and use of graphical means to help determine plausible transmission settings and transmission windows based on date of conversion and dates of positive culture. Among subjects whose clinic visits overlap (overlapping exposure windows), degree of physical closeness will be determined by evaluating locations that patients were at during their stay. Overall composite proximity measures will be determined that combine overlap time and multiple measures of physical closeness. Qualitative measures of proximity, such as location type (testing laboratory, patient room) will also be assessed. The probability of observing a number of subjects with the same strain in the same area and/or time by chance will be calculated, which will help indicate whether transmissions were more likely by one source or another (e.g., patient-to-patient or environment-to-patient). Several testing approaches will be used to compare the relationship between similarity of strains and degree of proximity of subjects. In the simplest form, descriptive statistics will be computed for proximity variables, for subjects who are determined to have similar strains versus those who have dissimilar strains, and multivariate or 2-sample *t*-tests (or equivalent nonparametric tests) will be performed to test for differences between these groups. More advanced modeling will be performed to determine the relationship between the degree of similarity in strains with the proximity and subject characteristic variables; multiple linear regression will be used when considering continuous strain similarity, while multiple logistic regression will be used when considering subjects with dichotomized (similar/dissimilar) strains to other subjects.

#### Analysis of secondary outcomes

The secondary endpoints will be to characterize CF Care Center adherence to the CF IP&C guidelines as well as points of common exposures or overlapping physical settings among patients. Basic summaries of points of potential transmission overlap will be provided. The incidence and prevalence of NTM species and subspecies within healthcare dust and water biofilms will be characterized. Comparisons of NTM dust and biofilm isolates and highly-related patient isolates will be provided.

#### Interim analyses

Review of interim data analyses will occur semi-annually.

## Discussion

The healthcare-associated links in transmission of NTM among pwCF (HALT NTM) study is a parallel multi-site study of pwCF within a single CF Center who have respiratory NTM isolates identified in clusters. This study facilitates standardizing epidemiologic investigation of healthcare-associated NTM outbreaks in U.S. CF Care Centers. CF Care Centers identified as having a possible NTM outbreak among pwCF based on membership in a genetically highly-related NTM cluster are eligible to participate. The study objective is to implement a standardized epidemiologic investigation in CF Care Centers identified as having clusters of highly similar NTM isolates among pwCF receiving care in a single center and to determine if CF Care Center healthcare environmental isolates (dust and water biofilms) are related to respiratory isolates recovered from pwCF. The HALT NTM study facilitates a structured process by which individual CF Care Centers perform epidemiologic evaluation of patients identified by the CO-NRC as part of an NTM cluster. The HALT NTM study is available to the entire CF Foundation Care Network, under a collaborative agreement, to conduct a standardized, independent, and confidential NTM outbreak investigation. This novel approach will provide opportunities to characterize potential transmission settings and sources of healthcare-associated acquisition of NTM by standardizing the epidemiologic investigation of clusters of NTM recovered from pwCF cared for at the same CF Care Center, thereby revealing risk factors for NTM acquisition.

Currently, WGS is the gold standard for NTM isolate comparison. However, the use of WGS to identify clonal strains of NTM is a new, rapidly evolving field. Evolutionary drift is expected to lead to an accumulation of SNPs within the genome observed in longitudinal samples from the same patient, such that it may be difficult to define a clonal group. The mutation rate, leading to SNP variation among samples in different individuals or the environment may be variable, and a definitive threshold for sameness/uniqueness may be context dependent. Fortunately, the SNP threshold defined by the CO-NRC compares directly to the threshold used by Bryant et al. [10, 13] and is applied to the evaluation of clusters within the CO-NRC WGS data [27]. The detailed molecular science and analysis to identify existence of NTM clusters makes it challenging to convey these types of results to a broad clinical audience who may not be familiar with the technology and analysis. We plan to address this knowledge gap by providing clear and concise details on clustered NTM findings.

Dust and biofilms from healthcare water supplies will be collected and NTM recovered, identified, and sequenced to determine if the respiratory NTM strain genotype among pwCF is similar to those recovered from the healthcare environment. The decision to sample water biofilms rather than free water was based on the fact that hydrophobic NTM cells prefer surface adherence over remaining suspended in water resulting in more concentrated NTM numbers in biofilms (1,000–15,000 CFU/cm2) compared to water (10–100 CFU/mL) [38]. Additionally, clinically-relevant NTM readily form biofilms on common plumbing materials [39]. There is also good evidence that individual NTM clones are stable in biofilms over long periods of time, including in hospital sources. One study reported that NTM from water biofilms have been repeatedly isolated over a 41-month period from a recirculating hospital hot water system [40]. Dust and biofilms from water supplies will be collected once, at the beginning of the study. Additional longitudinal environmental sampling is beyond the scope of the study, but may warrant further investigation in the future. HALT NTM standardizes healthcare-associated environmental dust and water biofilm collection and assess if hospital and clinic dust and/or plumbing sources could pose a risk to pwCF. This study provides a unique opportunity to characterize clinically-relevant NTM from environmental isolates in healthcare dust and water biofilms nationwide and serve as a platform for characterizing and describing the most common environmental sources within healthcare settings harboring clinically-relevant NTM isolates.

A standardized approach to investigate healthcare-associated NTM acquisition among pwCF is critical to better understanding prevention of infection. Collecting data on potential healthcare-associated transmission events in a systematic and uniform format will help build a statistical model to predict instances of increased probability of transmission events. Understanding risk for NTM transmission in the setting of the current IP&C guidelines for CF has the potential to improve our understanding of risk factors for NTM acquisition in the healthcare setting [37]. This study may provide data to better inform future updates to IP&C guidelines, thus significantly decreasing risk of healthcare-associated transmission events of NTM among pwCF.

When performing a multicenter epidemiological investigation, we may be faced with low enrollment, resulting in less than the anticipated 50% participation rate within CF Centers identified with highly similar strains of NTM. Preliminary discussions concerning willingness of the CF Care Center Program Directors to utilize the HALT NTM toolkit has been overwhelmingly positive and enrollment up to this point has been steady. We anticipate that the multi-site nature of the study will increase representation of geographic locations and represent diversity in sizes and patient populations of CF Care Centers throughout the U.S.

We acknowledge that the HALT NTM study includes culture and WGS evaluation of specific healthcare-associated sources of dust and plumbing biofilms but excludes WGS evaluation of other healthcare-associated environmental sources, such as water and soil. Clinically-relevant NTM has been cultured from environmental dust in homes of patients with NTM [41]. This is the first study, to our knowledge, that compares healthcare-associated environmental dust to clinical NTM isolates. The HALT NTM toolkit excludes evaluation of home and other outside environmental sources of NTM acquisition among patients with highly similar NTM strains within a single Center. While additional healthcare environmental sampling, as well as home environmental sampling, are both interesting and important, such sampling is beyond the scope of this study.

The HALT NTM study is currently enrolling CF Care Centers at a steady rate with 25% of eligible sites being enrolled within the first 1.5 years of the 3-year study (HALT NTM, NCT04024423). This novel study will standardize and broaden the scope of independent CF NTM outbreak investigations and will demonstrate the frequency and nature of healthcare-associated NTM transmission in U.S. CF Care Centers nationwide. Furthermore, it will provide valuable insights into modeling risk factors associated with healthcare-associated NTM transmission and better inform future IP&C guidelines for CF Care Centers. The HALT NTM study will also be the first of its kind to systematically characterize clinically-relevant NTM isolates of CF healthcare environmental dust and water biofilms and set the stage to describe the most common environmental sources within the healthcare setting harboring clinically-relevant NTM isolates.
